# Differential *in vivo* labeling with barcoded antibodies allows for simultaneous transcriptomic profiling of airway, lung tissue and intravascular immune cells

**DOI:** 10.3389/fimmu.2023.1227175

**Published:** 2023-11-29

**Authors:** Barbara C. Mindt, John Kim, Troy Warren, Yang Song, Antonio DiGiandomenico

**Affiliations:** ^1^ Vaccines & Immune Therapies, Biopharmaceuticals R&D, AstraZeneca, Gaithersburg, MD, United States; ^2^ Biologics Engineering, Oncology R&D, AstraZeneca, Gaithersburg, MD, United States

**Keywords:** respiratory disease, *in vivo* antibody labeling, spatial transcriptomics, scRNA-seq, bacterial lung infection, neutrophils

## Abstract

Single-cell RNA sequencing (scRNA-seq) is the state-of-the-art approach to study transcriptomic signatures in individual cells in respiratory health and disease. However, classical scRNA-seq approaches provide no spatial information and are performed using either bronchoalveolar lavage fluid (BAL) or lung single cell suspensions to assess transcript levels in airway and tissue immune cells, respectively. Herein we describe a simple method to simultaneously characterize transcriptomic features of airway, lung parenchymal and intravascular immune cells based on differential *in vivo* labeling with barcoded antibodies. In addition to gaining basic spatial information, this approach allows for direct comparison of cells within different anatomical compartments. Furthermore, this method provides a time- and cost-effective alternative to classical scRNA-seq where lung and BAL samples are processed individually, reducing animal and reagent use. We demonstrate the feasibility of this approach in a preclinical mouse model of bacterial lung infection comparing airway, parenchymal and vasculature neutrophils early after infection.

## 1 Introduction

Acute and chronic lung diseases are a leading cause of death and disability worldwide. Chronic obstructive pulmonary disease (COPD) alone accounted for more than 3.23 million deaths in 2019 representing the 3^rd^ leading cause on a global scale (1). On the other hand, lower respiratory infections (LRIs) including pneumonia and bronchiolitis are still a substantial cause of mortality in the elderly population and young children, respectively (2, 3). To counteract this trend and identify new therapeutic targets to tailor novel more effective intervention strategies, it is crucial to understand disease-associated changes in lung immune cells. Constant advances in single-cell RNA sequencing (scRNA-seq) technologies such as cellular indexing of transcriptomes and epitopes by sequencing (CITE-seq) which uses oligonucleotide-labeled antibodies now enable simultaneous analysis of gene as well as surface protein expression on a single cell level (4). However, scRNA-seq data provide no spatial information, which is especially problematic when analyzing immune responses in functionally highly compartmentalized organs such as the lung where expression profiles vary substantially based on whether cells are localized in the airways, the parenchyma or the vasculature.

Here, we developed a simple method to differentially label airway and blood immune cells by intravenous and intratracheal administration of distinct barcoded pan-leukocyte anti-CD45 antibodies. Single cell suspensions of lung and airway cells are further labeled with additional CITE-seq antibodies and transcript and cell surface protein expression levels are obtained following a generic CITE-seq workflow. Airway and intravascular leukocytes can be further stratified based on their respective anti-CD45 barcode, whereas parenchymal immune cells remain unlabeled. We demonstrate that antibody staining is highly specific for the respective compartment with minimal inter-anatomical cross-contamination. Using this basic spatial separation, we further show how neutrophil gene expressions profiles differ in the airways, tissue and vasculature following infection with non-typeable *Haemophilus influenzae* (NTHi), a bacterium often associated with exacerbations of chronic airway disease.

This method has a wide scope of applications and can be employed to analyze homeostatic differences in transgenic animal models as well immune responses in pre-clinical mouse models of lung disease including allergic airway inflammation, bacterial and viral lung infection as well as sterile injury models. The protocol is simple, adaptable and allows for simultaneous analysis of parenchymal, airway and blood immune cells from the same animal, thereby saving time and reagents.

## 2 Materials and equipment

### 2.1 Reagents

- NTHi glycerol stock
- Chocolate agar (Hardy Diagnostics; cat# E14)
- Phosphate buffered saline (PBS) pH 7.2 (Gibco, cat# 20012-043)
- KetaVed^®^ Ketamine Hydrochloride injection (100 mg/mL, VedCo Inc.)
- AnaSed^®^ Xylazine injection (20 mg/mL, Akorn Animal Health)
- Sterile saline (0.9%; Covetrus, cat# 069169)
- Dulbecco’s phosphate buffered saline (DPBS) (Gibco, cat# 14190-144)
- heat-inactivated fetal bovine serum (HI-FBS) (Gibco, cat# 10082-147)
- RPMI-1640 + L-Glutamine (Gibco, cat# 11875-085)
- recombinant murine CD45 (rmCD45) (100 μg/ml in DPBS; R&D, cat# 114-CD-050)
- Liberase™ TM (4 mg/mL in DPBS; Roche, cat# 5401127001)
- dsDNAse (ThermoFisher, cat# EN0771)
- ACK Lysing buffer (Gibco, cat# A10492-01)
- 0.4% Trypan Blue Stain (Gibco, cat# 15250-061)
- 0.5 M EDTA (Invitrogen, cat# 15575-038)
- 10% Bovine Serum Albumin (BSA) solution (Sigma-Aldrich, cat# A1595-50ML)
- Mouse anti-Ly-6G microbeads (Miltenyi Biotec, cat# 130-120-337)
- TruStain FcX™ (anti-mouse CD16/32) Antibody (BioLegend, cat# 101320)
- LIVE/DEAD™ Fixable Blue Dead Cell Stain Kit (Invitrogen, cat# L23105)
- eBioscience™ FoxP3/Transcription Factor Staining Buffer Set (Invitrogen, cat# 00-5523-00)

### 2.2 Equipment and consumables

- Sterile cotton tipped applicators (Dukal Corporation, cat# 9016)
- CO_2_ incubator
- PIPETBOY acu 2 pipet aid (Integra Biosciences)
- Serological pipettes (25 mL, 10 mL, 5 mL)
- Single-channel Micropipettes (P1000, P200, P10, P1; Gilson) + tips
- UV-Vis Scanning Spectrophotometer (GENESYS 10; Thermo Scientific)
- Disposable polystyrene cuvettes (ThermoFisher Scientific, cat# 221S)
- Heat Lamp and mouse restraint for i.v. injections
- 1 ml TB syringe 27G x 1/2 (BD, cat# 309623) for i.v. injections
- 1 ml TB syringe 25G x 5/8” (BD, cat#309626) for i.p. injections
- Dissection tools (tweezers, scissors)
- Gauze sponges (Dukal Corporation, cat# 4162)
- i.v. catheters (20G x 1”; Terumo, cat# SR*FF2025)
- 1 mL syringes (BD, cat# 309659)
- 500 mL Vacuum Filter/Storage Bottle Systems, 0.22 µm pore size (Corning, cat# 431097)
- 250 mL Vacuum Filter/Storage Bottle System, 0.22 µm pore size (Corning, cat# 431096)
- GentleMACS™ C tubes (Miltenyi Biotec, cat# 130-093-237)
- GentleMACS™ Octo Dissociator (Miltenyi Biotec, cat# 130-096-427)
- 50 mL Centrifuge tubes (VWR, cat# 89039-662)
- 70 μM cell strainers (Corning, cat# 431751)
- 40 μM cell strainers (Corning, cat# 431750)
- 3 mL syringes (BD, cat# 309657)
- 15 mL Centrifuge tubes (VWR, cat# 89039-670)
- Vi-CELL™ XR Cell Viability Analyzer (Beckman Coulter)
- Hemacytometer (Hausser Scientific, cat# 3100)
- QuadroMACS™ Separator (Miltenyi Biotec, cat# 130-090-976)
- MACS MultiStand (Miltenyi Biotec, cat# 130-042-303)
- LS columns (Miltenyi Biotec, cat# 130-042-401)
- Microcentrifuge (5427 R; Eppendorf)
- 96-well v-bottom microplates (Corning, cat# 3897)
- Chromium Next GEM Single Cell 3’ Kit v3.1 (10x Genomics, cat# 1000269)
- Dual Index Kit TT Set A (10x Genomics, cat# 1000215)
- Dual Index kit NT Set A (10x Genomics, cat# 1000242)
- NovaSeq 6000 SP Reagent Kit v1.5 (cat# 20028319)
- BD FACSymphony™ A5 cell analyzer (BD Biosciences)
- Chromium Controller (10x Genomics)
- NovaSeq 6000 Sequencing System (Illumina)

### 2.3 Solutions

- Ketamine (67 mg/kg) + xylazine (13 mg/kg) for anesthesia

 o 1.68 mL ketamine (100 mg/mL)
 o 1.63 mL xylazine (20 mg/mL)
 o 21.7 mL isotonic saline
 o Prepare fresh before use.

- Ketamine (500 mg/kg) + xylazine (50 mg/kg) for euthanasia

 o 1.6 mL ketamine (100 mg/mL)
 o 0.8 mL xylazine (20 mg/mL)
 o 0.8 mL isotonic saline
 o Prepare fresh before use.

- FACS buffer (DPBS + 2% FBS)

 o 10 mL HI-FBS
 o 490 mL DPBS
 o Sterile filter and store at 4°C

- PBE buffer (PBS + 0.5% BSA + 2 mM EDTA)

 o 25 mL 10% BSA solution
 o 2 mL 0.5 M EDTA
 o 473 mL PBS (pH7.2)
 o Sterile filter and store at 4°C

- Staining buffer (PBS + 1% BSA)

 o 20 mL 10% BSA solution
 o 180 mL DPBS
 o Sterile filter and store at 4°C

## 3 Methods

All procedures described in this study were performed in accordance with federal, state and institutional guidelines and were approved by the AstraZeneca Institutional Animal Care and Use Committee. Procedures were carried out using adult female Balb/cJ mice purchased from Taconic Biosciences (Germantown, NY). Animals were maintained under specific pathogen-free conditions with *ad libitum* access to food and water.

### 3.1 Preparation of bacterial inoculum and intranasal infection

- The day before infection, streak bacteria from frozen glycerol stock as a lawn on a nutrient agar plate using a sterile cotton swab. Incubate overnight at the requisite growth temperature. In our case NTHi was plated on chocolate agar and grown at 37°C in the presence of 5% CO_2_.
- Collect bacteria from the plate using a sterile cotton swab and suspend in sterile PBS (pH 7.2) to an optical density at 600 nm (OD_600_) of 1.0 which corresponds to a known amount of colony forming units (CFU)/mL.
- If necessary, dilute bacterial suspension further with PBS (pH 7.2) to the desired CFU/mL. Keep inoculum at room temperature and use within 15 min of preparation.
- Anesthetize mice by intraperitoneal injection of 200 μL ketamine/xylazine solution until a proper plane of anesthesia is attained as assessed by absence of pedal withdrawal reflex.
- Hold fully anesthetized mouse vertically and administer a total of 50 μL bacterial suspension dropwise in the middle of the nostrils using a P200 pipettor. Wait for mice to completely inhale the liquid before administering the next drop.

### 3.2 *In vivo* antibody labeling and lung isolation

- To label intravascular leukocytes, administer 150 μL (2 μg/mouse diluted in sterile DPBS) of anti-CD45 (clone 30-F11) intravenously via tail vein injection and let antibody circulate for 5 min.
- Euthanize animals by intraperitoneal injection of 200 μL concentrated ketamine/xylazine. Alternatively, a lethal dose of sodium pentobarbital can be used.
- Note: It is vital that lung barrier remains intact during euthanasia to avoid entry of blood in the alveoli and thereby mislabeling of blood leukocytes by the intratracheally administered pan-leukocyte antibody and *vice versa*. Consequently, euthanasia by carbon dioxide asphyxiation should be avoided since it may lead to alveolar extravasation, pulmonary oedema, and hemorrhage (5, 6). Also, refrain from cervical dislocation as a method of euthanasia to preserve the integrity of the trachea and adjacent neck structures.
- Pin mouse on its back on an angled (approximately 45°) dissecting tray and disinfect the neck area with 70% ethanol.
- Lift skin using forceps and make an incision at the mid-line of the neck with anatomical scissors.
- Use two fine-tipped forceps to carefully pull the salivary glands covering the trachea to the side. If blood vessels are damaged, use gauze pads to absorb blood before proceeding.
- Pull the muscle surrounding the trachea apart using tweezers and insert an i.v. catheter between the cartilage rings below the larynx. To avoid lung damage do not insert the catheter too far into the trachea.
- To label airway immune cells, carefully remove the puncture needle and slowly instill 800 μL cold anti-CD45.2 (clone 104; 2 μg/mouse diluted in sterile RPMI-1640) via the catheter using a 1 mL syringe.
- Leave the syringe attached to the catheter for 5 min before retrieving BAL.
- Transfer BAL to a GentleMACS C tube on ice containing 600 μL RPMI-1640 medium, 125 μL HI-FBS and 50 μL rmCD45 (100 μg/mL in PBS). Swirl to mix.
- Instill lungs once more with 800 μL cold RPMI-1640 + 50 μL rmCD45. Wait for 5 min, retrieve liquid and pool with the first lavage in the GentleMACS tube.
- Optional: Add appropriate volumes of TruStain FcX™ (anti-mouse CD16/32) antibody to the instillation mix and additionally to the pooled cells in the GentleMACS tube to prevent potential binding of rmCD45-anti-CD45 complexes to Fcγ receptors on respective cells. TruStain FcX™ should be used at 1 μg per 10^6^ cells. Adjust volume based on expected cell numbers in BAL and whole lung accordingly.
- Adjust volume in the GentleMACS to 2.35 mL with cold RPMI-1640.
- For lung isolation lift skin and peritoneal wall using forceps and make an incision beneath the ribcage. Cut the skin and peritoneal wall along the ribcage towards both sides of the abdomen without damaging underlying organs.
- Pierce and cut the diaphragm along the ribcage on both sides avoiding the lung.
- Cut the ribcage on both sides and remove the sternum and isolate lung from pleural cavity.
- Transfer to the GentleMACS tube containing the pooled BAL fractions.

### 3.3 Preparation of single cell suspensions from lung tissue

- Attach tubes on the GentleMACS dissociator and mince lung tissue using program “m_lung_01_02” (36 sec, 165 rpr).
- Add 125 μL Liberase™ (4 mg/mL in DPBS) and 3 μL dsDNAse, mix and digest for 30 min at 37°C (5% CO_2_).
- Further homogenize digested tissue on the GentleMACS dissociator using program “m_lung_02_01” (37 sec, 2079 rpr).
- Strain homogenate over a 70 μM cell strainer into a 50 mL Falcon tube
- Rinse cell strainer with 10 mL cold FACS buffer and centrifuge (5 min, 450 x g, 4°C).
- Remove supernatant, suspend pellet in 10 mL cold FACS buffer and centrifuge (5 min, 450 x g, 4°C).
- Remove supernatant, suspend pellet in 2 mL ACK lysing buffer and incubate for 4 min at room temperature to lyse red blood cells.
- Bring volume to 15 mL with FACS buffer, centrifuge (5 min, 450 x g, 4°C) and remove supernatant.
- Suspend pellet in 10 mL FACS buffer, centrifuge (5 min, 450 x g, 4°C), pipet off supernatant completely and resuspend cells in 1 mL staining buffer (PBS + 1% BSA)
- Dilute cells 1:10 in PBS (100 μL cells + 900 μL PBS) and determine viable cell counts using an automated cell counter. Alternatively, dilute cells in Trypan Blue solution and count manually with a hemacytometer according to the manufacturer’s instructions.
- Transfer 3 x 10^6^ cells/sample to each well of a 96-well v-bottom plate for FACS staining.

### 3.4 Neutrophil magnetic bead enrichment

Neutrophils are isolated using anti-Ly-6G microbeads according to the manufacturer’s instructions.

- Strain cells over a 70 μM cell strainer and wash strainer with 5 ml cold PBE buffer.
- Centrifuge cells at 300 x g for 10 min and completely remove supernatant.
- Suspend cell pellet in 90 μL cold PBE buffer per 1 x 10^7^ cells and add 10 μL anti-Ly-6G microbeads per 1 x 10^7^ cells. Adjust buffer and bead volumes accordingly.
- Mix and incubate at 4°C (no for 10 min.
- Add 2 mL cold PBE buffer/10^7^ cells and centrifuge for 10 min at 300 x g.
- During the spin, load an LS column in the QuadroMACS™ Separator attached to the MACS MultiStand and equilibrate with 3 mL cold PBE buffer.
- Discard supernatant and suspend up to 10^8^ cells in 500 μL cold PBE buffer.
- Load cell suspension on LS column and wait for column reservoir to empty by gravity flow.
- Wash column 3x with 3 ml cold PBE buffer, remove column from magnet and transfer to a 15 mL centrifugation tube.
- Add 5 ml cold PBE buffer and immediately force liquid through column using the provided plunger.
- Spin cells at 300 x g for 10 min, suspend in 1 mL cold FACS buffer and determine viable cell counts using an automated cell counter or count manually with a hemacytometer and Trypan Blue solution according to the manufacturer’s instructions.

### 3.5 Cell surface protein labeling with TotalSeq-B antibodies for CITE-seq analysis

- Spin cells and suspend in 50 μl staining buffer per 10^6^ cells.
- Transfer 150 μl sample to the wells of a 96-well v-bottom plate for FACS staining and keep on ice (see 3.6).
- Transfer 50 μl cell suspension to a 1.5 mL Eppendorf tube and add 5 μl TruStain FcX blocking reagent.
- Mix and incubate for 10 min at 4°C to block FcγRII/III receptors.
- Add 45 μl TotalSeq-B antibody mix prepared in staining buffer (0.5 μg antibody/sample). All antibodies used in the mix are listed in **Supplementary Table 1**.
- Incubate for 30 min at 4°C and centrifuge (5 min, 350 x g, 4°C).
- Discard supernatant and wash cell pellets 3x with 1 mL staining buffer.
- Resuspend in 300 μl PBS and determine cell counts with a hemacytometer.
- Dilute to 700 - 1200 cells/μL (= 0.7 – 1.2 x 10^6^ cells/mL).

### 3.6 Cell surface protein labeling for flow cytometry

- Transfer 1-5 x 10^6^ cells/sample to the wells of a 96-well v-bottom plate, centrifuge (5 min, 450 x g, 4°C) and discard supernatant.
- Resuspend cells in 50 μL cold CD16/CD32 dilution (1:100 in FACS buffer) and incubate for 15 min on ice to block FcγRII/III receptors.
- While cells are blocking prepare antibody cocktail in cold FACS buffer (antibodies used and dilutions are provided in **Supplementary Table 1**.
- Centrifuge (5 min, 450 x g, 4°C), discard supernatant, resuspend cell pellet in 50 μL antibody dilution and incubate for 30 min on ice in the dark.
- Add 120 μl cold DPBS and spin (5 min, 450 x g, 4°C).
- Remove supernatant, resuspend cells in 150 μl cold DPBS and spin again (5 min, 450 x g, 4°C).
- While cells are spinning, resuspend Fixable Blue viability dye in anhydrous DMSO according to the manufacturer’s instructions, further prepare a 1:1000 dilution in cold DPBS and store in the dark on ice until use.
- Remove supernatant, resuspend cell pellet in 50 μl viability dye dilution and incubate on ice in the dark for 30 min.
- Add 120 μl cold FACS buffer, spin (5 min, 450 x g, 4°C) and remove supernatant.
- Resuspend cells in 150 μl cold FACS buffer, spin again (5 min, 450 x g, 4°C).
- During spin, prepare Fix/Perm solution of FoxP3/Transcription Factor Staining Buffer Set according to the manufacturer’s protocol (1 part concentrate + 3 parts diluent).
- Discard supernatant, resuspend cells in 50 μl Fix/Perm solution and incubate in the dark on ice for 30 min.
- Spin (5 min, 450 x g, 4°C), remove supernatant and resuspend pellet in 150 μl cold FACS buffer.
- Centrifuge again (5 min, 450 x g, 4°C) and add 150 μl cold FACS buffer.
- Cover plate with a sealing film, wrap in tin foil and store in the fridge for up to three days until acquisition on a flow cytometer.

### 3.7 CITE-seq

Labeled cell suspensions were partitioned into single-cell droplets with a Chromium Controller (10x Genomics) and gene expression and cell surface protein libraries were further generated using Chromium Single Cell 3’ Reagent Kits with Feature Barcoding Technology (10x Genomics) following the manufacturer’s recommendations. Samples were sequenced on a NovaSeq 6000 Sequencing System (Illumina) and the resulting FASTQ files were processed with CellRanger (version 6.0.1, 10X Genomics). Unless noted, all the analysis for the single-cell RNA-sequencing was done using the Python Scanpy framework (Scanpy version 1.9.1) (7). Cells were further filtered to include cells with log1p values of isotype control antibody reads (< 6), number of antibodies detected per cell (< 30), mitochondrial reads (< 30%), total RNA reads (< 40000), number of genes (< 6000 and > 200). To normalize antibody reads in the dataset, denoised and scaled by background (DSB) program (8) using the default parameters implemented in MUON package (version 0.1.2) was used (9). Filtered raw RNA reads were further normalized and log-transformed using *normalize_total* and *log1p* functions, respectively and data were further scaled using *scale* function. Before data scaling, log-normalized data were stored separately for further downstream analysis such as differential expression test. DSB-normalized protein reads were clustered using Leiden algorithm after generating a neighborhood graph with default parameters and for three different resolution settings (0.25, 0.5, 0.75). For each resolution, a heatmap was plotted for the average expression of proteins per cluster to determine at which resolution biologically relevant clusters can be visualized. To assign the respective anatomical location to each cell, we first used histograms of DSB-normalized CD45 and CD45.2 antibody reads to determine the cut-offs. Respective cut-offs were applied to each separate anatomical location (0.3 for CD45.2^+^CD45^-^ airway cells; > 0.28 for CD45.2^-^CD45^+^ intravascular leukocytes; < 0.3 and < 0.28 for CD45.2^-^CD45^-^ parenchymal cells). For differential expression (DE) test per cluster, *tl.rank_genes_groups* function with Wilcoxon rank-sum test was used.

### 3.8 Statistical analyses

All data were analyzed with GraphPad Prism software (GraphPad Software). P values below 0.05 were regarded as statistically significant (*p<0.05, **p<0.01, ***p<0.001). Unless otherwise indicated, figures display means ± standard deviation (SD). Experiment sample sizes (n), experiment replicate numbers and statistical tests used are included in the respective figure legends.

## 4 Results

### 4.1 Administration of i.v. and i.t. anti-CD45 antibodies results in efficient and stable labeling of intravascular and airway immune cells

**Figure 1:**
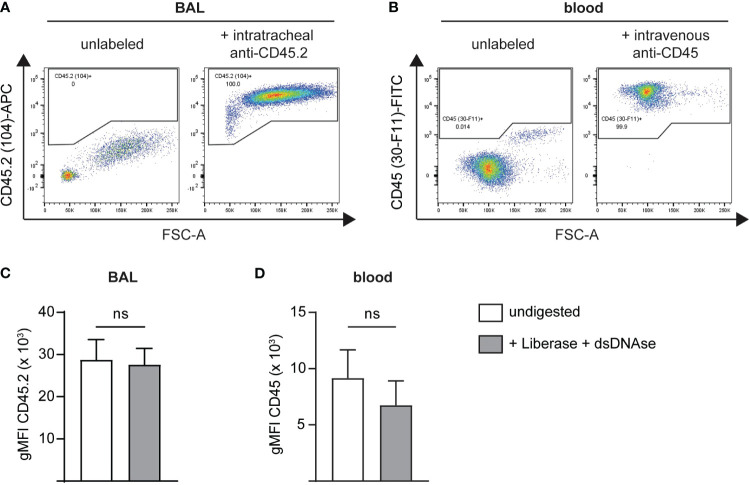
Administration of i.v. and i.t. anti-CD45 antibodies results in efficient and stable labeling of intravascular and airway immune cells. BAL or blood cells were *in vivo* labeled, isolated and co-stained with an anti-CD45 antibody binding to a different CD45 epitope followed by flow cytometric analysis. Representative flow cytometry plots of single live CD45^+^ **(A)** airway cells after intratracheal administration of 2 μg anti-CD45.2 (clone 104)-APC followed by BAL cell staining with anti-CD45 (clone 30-F11)-FITC or **(B)** blood leukocytes following intravascular administration with 2 μg anti-CD45 (clone 30-F11)-FITC via tail vein injection and staining of blood cells with anti-CD45.2 (clone 104)-APC. **(C)** gMFI of CD45.2 (clone 104) on BAL cells before (white bar) and after digestion (grey bar) with Liberase and dsDNAse. **(D)** gMFI of CD45 (clone 30-F11) on blood cells before (white bar) and after digestion (grey bar) with Liberase and dsDNAse. Data are representative of three independent experiments with n = 3–5 mice per group. Data are shown as mean ± SD and significance between groups was determined using a Mann-Whitney test. i.v., intravenous; i.t., intratracheal; FITC, fluorescein isothiocyanate; APC, allophycocyanin; BAL, bronchoalveolar lavage; gMFI, geometric mean fluorescence intensity. ns, non-significant.

For *in vivo* labeling of immune cells in the vasculature and the airways, two different clones of pan-leukocyte anti-CD45 antibodies, clone 30-F11 and clone 104, are administered intravascularly or intratracheally, respectively. To ensure the amount of antibody used is sufficient to label all cells present in the respective anatomical compartment, we administered fluorescently labeled antibody versions via the designated route. In addition, we stained the labeled BAL or blood cells with the other anti-CD45 clone to gate on all CD45^+^ cells. Potential steric competition between antibodies was assessed beforehand by preincubation of mouse splenocytes with either fluorescent clone 104 or 30-F11 followed by staining with the respective other clone and comparison to single-stained cells (**Supplementary Figure 1**). 30-F11 staining intensity, depicted as geometric mean fluorescence intensity (gMFI), was not affected by prior labeling of cells with clone 104 (**Supplementary Figure 1A**). When cells were prelabeled with 30-F11, staining intensity of 104 was significantly lower compared to the single-labeled cells indicating some binding interference (**Supplementary Figure 1B**). However, frequencies of double-positive cells remained comparable to the 104-only stained control indicating both clones can be used for simultaneous labeling of leukocytes (**Supplementary Figures 1C, D**). Importantly, flow cytometric analysis following *in vivo* antibody instillation showed that all immune cells in the airway and vasculature were labeled with clone 104 or 30-F11 respectively, indicating that the used concentrations and experimental conditions were suitable to efficiently tag leukocytes *in vivo* (**Figures 1A, B**). Studies were performed on infected as well as uninfected animals to ensure consistent labeling under both conditions and antibody concentrations should be tested beforehand when a different model is used. To determine whether CD45 labeling remains stable during downstream processing steps, we labeled airway and blood leukocytes with fluorescently labeled antibodies *in vivo*. Isolated BAL and blood cells further underwent enzymatic digestion with Liberase and dsDNase and CD45 gMFI was compared to that of undigested cells (**Figures 1C, D**). Double strand-specific DNase was used to preserve the integrity of the TotalSeq B-barcodes since regular DNase I also utilizes single-stranded DNA as a substrate. gMFIs did not change with digestion indicating that anti-CD45 antibodies remain stably bound to their epitopes during processing and can be confidently used under the tested conditions for scRNA-seq analysis.

### 4.2 Excess anti-CD45 antibodies can be effectively neutralized with recombinant CD45

**Figure 2:**
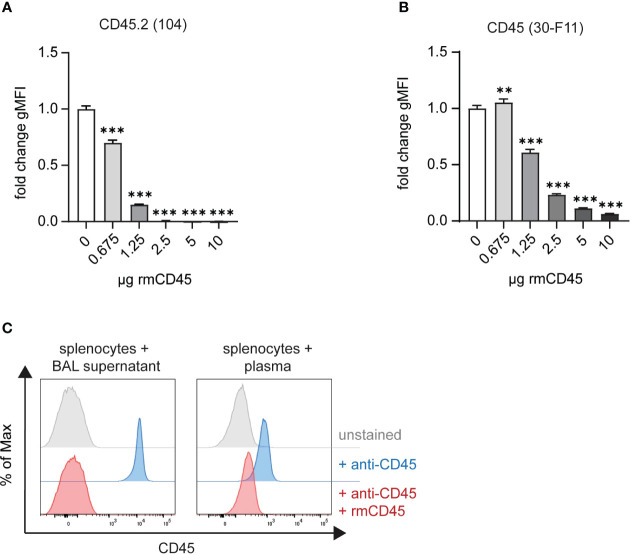
Excess anti-CD45 antibodies can be effectively neutralized with recombinant CD45. *In vitro* neutralization of 2 μg **(A)** anti-CD45.2 (clone 104)-APC or **(B)** anti-CD45 (clone 30-F11)-FITC with 0.675 – 10 ug rmCD45. Fluorochrome-labeled antibodies were incubated for 30 min with indicated amounts of rmCD45 in lung digestion medium and neutralization efficiency was assessed based on the antibody’s ability to stain naïve mouse splenocytes thereafter. Labeling efficiency is depicted as gMFI on single live cells. **(C)** Representative histograms plots of anti-CD45 *in vivo* neutralization in BAL (left panel) following consecutive i.t. administration of anti-CD45.2 (2 μg; clone 104)-APC and rmCD45 (10 μg) or in blood (right panel) after consecutive i.v. injection of anti-CD45 (2 μg; clone 30-F11)-FITC rmCD45 (10 μg). Neutralization efficiency was assessed by the ability of BAL supernatant or plasma to label naïve mouse splenocytes and was analyzed by flow cytometry. Unlabeled splenocytes or blood cells (grey histograms) were used as negative controls and mice that received antibody only (red histograms) served as maximum staining controls. Data are representative of three independent experiments with n = 3–5 mice per group. Data are shown as mean ± SD with **p < 0.01, ***p < 0.001 as determined by one-way ANOVA followed by Dunnett’s multiple comparisons test. rmCD45, recombinant murine CD45; i.v., intravenous; i.t., intratracheal; FITC, fluorescein isothiocyanate; APC, allophycocyanin; BAL, bronchoalveolar lavage; gMFI, geometric mean fluorescence intensity.

During downstream lung processing, the airway and intravascular boundaries are disintegrated, and excess antibodies need to be neutralized to avoid cross-labeling of blood cells with unbound airway anti-CD45 or *vice versa*. We therefore instilled recombinant murine CD45 (rmCD45) intratracheally after antibody labeling and added additional recombinant protein to the lung digestion medium. The amount of rmCD45 needed to neutralize potentially unbound antibody was assessed by titration experiments where we incubated the respective fluorescent antibodies with increasing concentrations of rmCD45. This was followed by addition of mouse splenocytes and analysis of CD45 labeling by flow cytometry. As expected, all splenocytes were stained with either antibody in the absence of rmCD45 (**Figures 2A, B**). Clone 104 staining was completely abolished with 2.5 µg rmCD45 indicating that all antibody in solution had been effectively neutralized (**Figure 2A**). Similar observations were made with clone 30-F11, albeit 10 µg of rmCD45 were needed to achieve a similar degree of neutralization (**Figure 2B**). We next assessed whether 10 µg rmCD45 was a suitable concentration to neutralize unbound antibodies *in vivo*. We first analyzed neutralization efficiency in the airways by consecutive intratracheal administration of fluorescent anti-CD45 (clone 104) and rmCD45. To assess neutralization of anti-CD45 (30-F11), we administered a fluorescent version via tail vein injection, allowed the antibody to circulate and injected rmCD45 via the same route. Neutralization efficiency was assessed by the ability of BAL supernatant or plasma to label naïve mouse splenocytes and was analyzed by flow cytometry (**Figure 2C**). As expected, when only anti-CD45 was administered, all splenocytes were labeled, whereas the presence of rmCD45 in either airway or blood completely neutralized unbound antibody, evident by the lack of splenocyte labeling. We therefore chose 10 µg rmCD45 to proceed with our evaluation. It should be noted that this is a reference value for our model system. Component concentrations for other *in vivo* models should be tested and confirmed empirically before RNA-seq analysis.

### 4.3 Intravascular and airway immune cells can be distinguished by compartmental CD45 labeling

**Figure 3:**
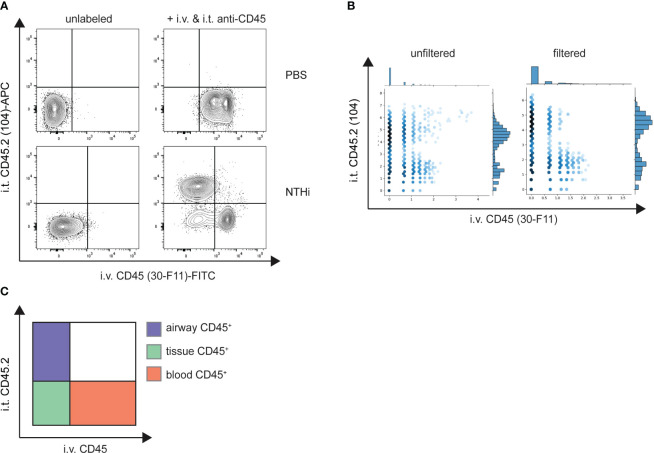
Intravascular and airway immune cells can be distinguished by compartmental CD45 labeling. Mice were intranasally infected with NTHi (5 x 10^7^ CFU/mouse) or PBS as a control. 48 h post infection mice were administered with 2 µg fluorescent **(A)** or 2 µg TotalSeq-B **(B)** anti-CD45 (clone 30-F11) and anti-CD45.2 (clone 104) intravenously or intratracheally, respectively. Excess antibodies were neutralized with 10 µg of rmCD45 and BAL and lungs were isolated. CD45 labeling on isolated neutrophils was analyzed by flow cytometry or scRNA-seq and designated as airway, blood or tissue cells based on their respective label **(C)**. **(A)** Representative flow cytometry plots of Ly6G^+^ neutrophils isolated from lungs of control PBS (top panel) or NTHi-infected mice (bottom panel). Unlabeled control mice were used to define positive populations (left). **(B)** Unfiltered (left) and filtered (right) hexagon plots showing the distribution of CD45 antibody barcodes on Ly6G^+^ neutrophils from infected mice. Non-single cells were filtered out based on antibody and RNA reads. Flow cytometry data are representative of two experiments with n = 3 – 5 mice per group. scRNA-seq was performed once. NTHi, non-typeable *Haemophilus influenzae*; i.v., intravenous; i.t., intratracheal; FITC, fluorescein isothiocyanate; APC, allophycocyanin.

To test the validity of our method we used an acute bacterial pneumonia model where we intranasally infected mice with a high dose of NTHi or administered PBS as a control. We additionally employed an acute LPS-induced lung inflammation model to further validate the specificity of our *in vivo* staining procedure. Blood and airway leukocytes were labeled with either fluorescent or TotalSeq-B anti-CD45 antibodies at 48 hours post-infection or 24 h after LPS challenge. Depending on the cell population of interest, enrichment is usually performed preceding scRNA-seq to increase sequencing resolution. This often entails positive selection for CD45^+^ leukocytes using magnetic bead enrichment. We therefore tested whether *in vivo* CD45 labeling would interfere with positive selection via bead-bound anti-CD45 antibodies using lung cells labeled with anti-CD45-FITC (clone 30-F11), anti-CD45.2-APC (clone 104) or a combination thereof. While the purity of the CD45^+^ fraction was comparable in all conditions (>97%) after enrichment, the flowthrough (CD45^-^ fraction) of cells previously labelled with anti-CD45 (clone 30-F11) contained significantly higher frequencies of CD45^+^ cells than the unstained control or cells pre-labeled with anti-CD45.2 (clone 104) (**Supplementary Figure 2A**). In accordance, numbers of CD45^+^ cells (**Supplementary Figure 2B**) were markedly lower in CD45^+^ fractions from 30-F11-stained cells as opposed to unstained or 104-stained cells. On the other hand, there was a >25-fold increase of CD45^+^ cells recovered in the flowthrough, indicating insufficient retention of 30-F11-stained cells in the column. CD45^+^ enrichment should therefore be avoided, and enrichment should be performed by negative selection or with magnetic beads of a different specificity. We therefore directly enriched for neutrophils with Ly6G microbeads and using fluorescently labeled antibodies showed they are almost exclusively found in the lung vasculature of uninfected animals (**Figures 3A, B**). As expected, inoculation with NTHi or LPS challenge resulted in neutrophil recruitment from the blood to the site of infection indicated by distinct populations of CD45.2(104)^+^CD45.2(30-F11)^-^ airway, and smaller populations of CD45.2(104)^-^CD45.2(30-F11)^+^ intravascular and CD45.2(104)^-^CD45.2(30-F11)^-^ parenchymal neutrophils (**Figures 3A, C**; **Supplementary Figure 5A**). Importantly, almost no double-positive cells were observed indicating the absence of cross-labeling. This can be attributed to efficient neutralization of excess antibodies since all cells were labeled with intratracheal anti-CD45 antibody in the absence of rmCD45 after NTHi infection (**Supplementary Figure 3**). No staining with intravascular antibody could be detected on Siglec-F^+^CD11c^+^ airway-resident alveolar macrophages after NTHi infection, confirming the absence of antibody cross-contamination under the indicated conditions (**Supplementary Figure 3C**). Distinct neutrophil populations could also be distinguished following infection based on distribution of CD45 barcodes after labeling with TotalSeq-B antibodies (**Figure 3B**) validating the compartmentalized *in vivo* labeling approach. Double positive signals exclusively stemmed from non-single cells and were filtered based on total antibody and RNA reads per cell. Specifically, cells that have detectable reads of 30 or more surface proteins used for labelling were deemed to be cell clumps or doublets and removed from the downstream analysis. In addition, cells with high RNA reads per cell (> 40,000) were also filtered out to remove low-quality or doublets from the analysis.

### 4.4 Neutrophils exhibit location-specific gene expression signatures after NTHi infection

**Figure 4:**
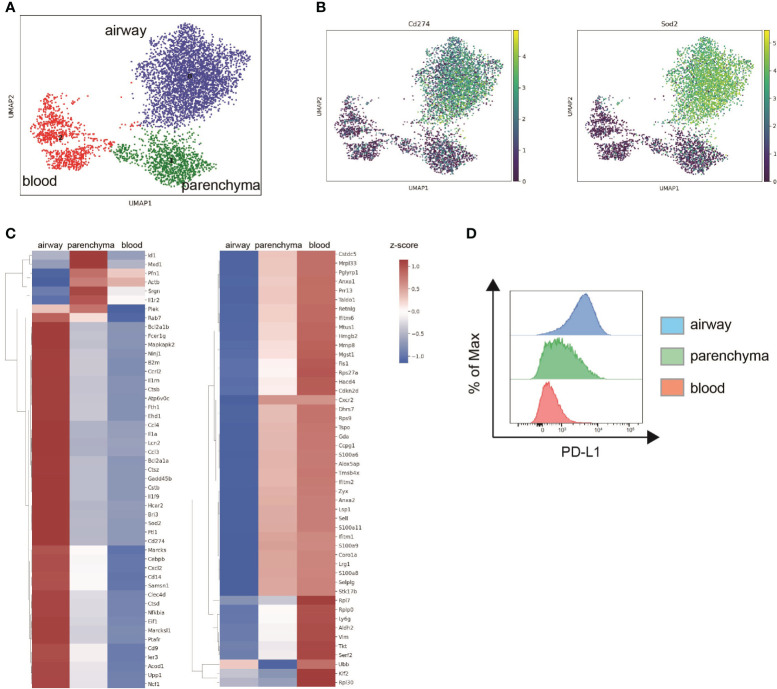
Neutrophils exhibit location-specific gene expression signatures after NTHi infection. Mice were intranasally infected with NTHi (5 x 10^7^ CFU/mouse) and administered with i.v. or i.t. anti-CD45 antibodies at 48 h post infection followed by scRNA-seq or flow cytometry analysis. **(A)** UMAP of 5,543 lung neutrophils subclustered based on CD45 surface labeling in airway (blue), parenchymal (green), and blood neutrophils (red). **(B)** UMAP of relative expression of Cd274 (PD-L1) (left) and Sod2 (right) in neutrophil subclusters. **(C)** Clustered heat maps depicting relative expression of top 30 upregulated (left) and downregulated genes (right) in airway and parenchymal neutrophils compared to blood neutrophils. Differentially expressed genes over blood neutrophils were determined using Wilcoxon test and genes differentially expressed in both airway and parenchyma are only displayed once. The log-normalized expression values were standardized (z-score) for visualization. **(D)** Representative flow cytometry histogram plot of PD-L1 cell surface expression on airway (blue), parenchymal (green), and blood neutrophils (red). Data are representative of one sequencing experiment **(A, B)** and two independent flow cytometry experiments **(C)** with n = 3–5 mice per group.

To analyze location-specific gene expression profiles, filtered scRNA-seq data were projected into two dimensions using Uniform Manifold Approximation and Projection (UMAP) based on their CD45 surface label. Unbiased clustering identified three distinct neutrophil subpopulations, cluster 0 (airway), cluster 2 (blood) and cluster 1 (parenchyma) (**Figure 4A**). Higher transcript levels of inflammatory and antibacterial neutrophil response genes such as *Sod2, Cd274*, *Ncf1*, *Lcn2*, *FceR1g* and the cytokines/chemokines *Il1a*, *Ccl3*, *Ccl4* and *Cxcl2* suggest increased activation of airway over tissue and blood subpopulations (**Figures 4B, C**). Downregulated genes in airway neutrophils including *Sell*, *Zyx*, *Selplg*, *Coro1a, S100a8, S100a9* and *Cxcr2* were mainly associated with neutrophil locomotion and adhesion (**Figure 4C**). In general, tissue and blood neutrophils were more similar in their expression profiles. In accordance, subclustering based on transcriptomic data alone and subsequent analysis of CD45 surface labeling in the resulting clusters showed that airway neutrophils are transcriptionally distinct while gene expression of blood and parenchyma neutrophils was partially overlapping (**Supplementary Figures 4A, B**). Like airway neutrophils, upregulated genes in blood and tissue neutrophils were involved in inflammatory and antibacterial responses (*Cd14, Acod1, Ccrl2, Cxcl2, Il1rn, Ncf1, Ptafr, Clec4d, B2m*) while downregulated genes were associated with cytoplasmic translation (*Rpl7, Rpl30, Rps9, Rps27a, Rplp0*) (**Figure 4C**). We further assessed PD-L1 surface expression in neutrophil populations by flow cytometry after *in vivo* labeling with fluorescent anti-CD45 antibodies. Surface PD-L1 was highly expressed on airway neutrophils, with intermediate and low levels found in lung parenchyma and blood neutrophils, respectively (**Figure 4D**). This finding was validated in an LPS-induced acute lung inflammation model showing high expression of PD-L1 on lung and parenchymal neutrophils while their blood counterparts only exhibited low PD-L1 levels (**Supplementary Figure 5B**). These findings correlated strongly with transcript levels after NTHi infection further validating our *in vivo* labeling approach for scRNA-seq.

## 5 Discussion

Herein we describe a simple, time- and cost-efficient protocol that allows for simultaneous transcriptomic analysis of mouse airway, lung tissue and blood leukocytes. This is especially important during lower respiratory tract infection and inflammation models where immune cells are rapidly recruited to the airways and adapt their gene expression profile to perform their respective effector functions. Spatial clustering based on compartmental antibody labeling allows not only for differential gene expression analysis within the same population, which may not be obvious on the whole-population level, but also to deduct trajectories across pseudotime. This method can be employed at steady state or in a wide array of pre-clinical respiratory disease models including pulmonary infection as well as sterile injury and allergic airway inflammation models. However, in models with extensive vascular leakage which may compromise the integrity of the staining it would be advised to perform appropriate controls such as intravenous Evans blue injection followed by lung histology.

Our method is highly adaptable and has only minor drawbacks that can be easily counteracted. Importantly, we demonstrate that *in vivo* labelling interferes with commercial magnetic bead CD45 positive selection kits, therefore other enrichment kits should be considered. It may also affect the binding of CD45 hashtag antibodies and hashing and demultiplexing should therefore be performed using antibodies against other ubiquitously expressed surface markers, such as H-2 MHC Class I. If only specific cell populations are of interest, *in vivo* labelling can also be performed with population-specific antibodies given the availability of more than one clone and non-overlapping epitope binding. This would again allow for leukocyte positive selection and the use of anti-CD45 hashtag antibodies.

Altogether, our approach builds upon existing scRNA-seq protocols by describing a simple method to simultaneously characterize transcriptomic features of airway, lung parenchymal and intravascular immune cells. We believe this approach is applicable to multiple disease models and will further aid in elucidating mechanisms of disease development and progression. In addition, this approach could help identify novel therapeutic targets or delineate disease-specific biomarkers.

## Data availability statement

The original contributions presented in the study are publicly available. This data can be found here: GEO, GSE246845. 

## Ethics statement

The animal study was approved by AstraZeneca Institutional Animal Care and Use Committee. The study was conducted in accordance with the local legislation and institutional requirements.

## Author contributions

BM designed the study, performed experiments and wrote the manuscript. JK analyzed scRNA-seq data and edited the manuscript. YS prepared libraries and performed sequencing. TW assisted with *in vivo* experiments. AD contributed to writing and editing. All authors contributed to the article and approved the submitted version.
