# Supplementary material for: Differential in vivo labeling with barcoded antibodies allows for simultaneous transcriptomic profiling of airway, lung tissue and intravascular immune cells
Source: Front Immunol. 2023 Nov 29;14:1227175. doi: 10.3389/fimmu.2023.1227175 (PMC10716273; doi:10.3389/fimmu.2023.1227175)
Supplement: Supplementary file 1 [file DataSheet_1.docx]

Supplementary Material

Differential *in vivo* labeling with barcoded antibodies allows for simultaneous transcriptomic profiling of airway, lung tissue and intravascular immune cells

Barbara C. Mindt^*^, John Kim, Troy Warren, Yang Song, Antonio DiGiandomenico

*** Correspondence:** Barbara C. Mindt: barbara.mindt@astrazeneca.com

# Supplementary Methods

## Generation of splenic and blood single cell suspensions.

Mouse spleens were isolated and stored in FACS buffer (DPBS + 2% HI-FBS) on ice until processing. To generate single cell suspensions, spleens were mashed through a 70 μM cell strainer (Corning, cat# 431751) into a 50 mL centrifugation tube using the plunger of a 3 mL syringe. Strainer was washed with 10 mL FACS buffer, cells were spun down (5 min, 450 x g, 4°C) and supernatant was removed. Cell pellet was suspended in 2 mL ACK Lysing buffer (Gibco, cat# A10492-01) and incubated for 5 min at room temperature to lyse red blood cells. 10 mL cold FACS buffer was added, cells were centrifuged (5 min, 450 x g, 4°C) and supernatant was discarded. Cells were washed again with 10 mL FACS buffer, suspended in 1 mL FACS buffer and stored on ice. 50 – 100 μl were used for flow cytometry staining.

Whole blood was collected by cardiac puncture or from the submandibular vein and stored in K_2_EDTA tubes (BD, cat# 365974) to prevent clotting. 100 μl blood was further transferred to an Eppendorf tube, 1 mL ACK Lysing buffer (Gibco, cat# A10492-01) was added and red blood cells were lysed for 5 min at room temperature. Cells were centrifuged (5 min, 450 x g, 4°C), supernatant was discarded, and pellet was washed twice with 1 mL FACS buffer. Cells were taken up in 200 μl FACS buffer and 50 - 100 μl were used for antibody staining.

## Plasma preparation.

Up to 500 μl whole blood was collected in K_2_EDTA tubes (BD, cat# 365974) by cardiac puncture and centrifuged (10 min, 2000 x g, 4°C). Plasma supernatant was transferred to an Eppendorf tube and stored in the dark on ice.

## Lung leukocyte enrichment.

Leukocytes were enriched from lung single cell suspensions using anti-CD45 microbeads (Miltenyi Biotec, cat# 130-052-301) according to the manufacturer’s instructions. Briefly, cell suspensions were strained over a 70 μM cell strainer to remove clumps and strainer was washed with 5 ml cold PBE buffer (PBS + 0.5% BSA + 2 mM EDTA). Cells suspensions were centrifuged (300 x g, 10 min, 4°C) and supernatant was discarded. Pellet was suspended in cold PBE buffer (90 μL per 1 x 10^7^ cells), anti-Ly-6G microbeads (10 μL per 1 x 10^7^ cells) were added and mix was incubated at 4 °C for 10 min. After incubation, cold PBE buffer (2 mL per 10^7^ cells) was added and centrifuged (10 min, 300 x g, 4 °C). Supernatant was discarded and up to 10^8^ cells were suspended in 500 μL cold PBE buffer. An LS column was loaded on the QuadroMACS^TM^ Separator and equilibrated with 3 mL cold PBE buffer. Cell suspension was loaded on column and column reservoir was allowed to empty by gravity flow. Column was washed 3x with 3 ml cold PBE buffer and wash fractions were collected (CD45^-^ fraction). After the last wash, column was removed from magnet and transferred to a 15 mL centrifugation tube. 5 ml cold PBE buffer were added, and liquid was immediately forced through the column using the provided plunger (CD45^+^ fraction). CD45^+^ and CD45^-^ fractions were spun at 300 x g for 10 min (4 °C) and suspended in 1 mL cold FACS buffer each. Viable cell counts were determined using an automated cell counter or a hemacytometer and Trypan Blue solution according to the manufacturer’s instructions. 50 - 100 μl were used for flow cytometry analysis.

## LPS-induced acute airway inflammation model.

6-8-week-old Balb/c mice were intranasally challenged with 0.3 mg/kg lipopolysaccharide (LPS, *E. coli* O111:B4; Sigma-Aldrich, cat# 437627) in 50 μl PBS. Fluorescently labeled anti-CD45 antibodies were intravascularly and intratracheally administered 24 h after challenge as described.

# Supplementary Figures and Tables

## Supplementary Figures

**Supplementary Figure 1: Anti-CD45 clones 30-F11 and 104 bind non-competitively to their target epitopes.** To assess whether clones 30-F11 and 104 can simultaneously bind to target cells, mouse splenocytes were pre-incubated with either fluorescent antibody followed by staining with the respective other clone. gMFI was analyzed on single viable cells by flow cytometry. **(A)** Representative flow cytometry plots of unstained (left panel), single- (middle panel) and double-stained cells (right panel). **(B-C)** CD45 gMFI on single- (white bar) or dual-labeled splenocytes (grey bar). **(D)** Frequencies of live double-positive cells after consecutive antibody labeling. Data are representative of two independent experiments with n = 3–5 mice per group. Data are shown as mean ± SD with ***p < 0.001 as determined by Mann-Whitney test. Abbreviations: gMFI, geometric mean fluorescence intensity.

**Supplementary Figure 2: Labeling with anti-CD45 (30-F11) interferes with CD45^+^ cell enrichment using mouse CD45 microbeads.** Lung single cell suspensions were labeled with anti-CD45-FITC (clone 30-F11), anti-CD45.2-APC (clone 104) or a combination thereof followed by enrichment for CD45^+^ cells using positive selection magnetic beads (Miltenyi Biotec). Cell numbers and purities of the resulting CD45^+^ and CD45^-^ fractions were assessed by flow cytometry. (**A**) Frequencies and (**B**) numbers of CD45^+^ cells in unenriched lung single cell suspensions as well as in CD45^+^ and CD45^-^ fractions after enrichment. Data are representative of one experiment with n = 1 sample per group.

**Supplementary Figure 3: Intravascular and airway immune cells can be distinguished by compartmental CD45 labeling.** Mice were intranasally infected with NTHi (5 x 10^7^ CFU/mouse) or PBS as a control. 48 h post infection mice were administered with 2 µg fluorescent (**A**) or 2 µg TotalSeq-B (**B**) anti-CD45 (clone 30-F11) and anti-CD45.2 (clone 104) intravenously or intratracheally, respectively. Isolated lungs and BAL cells were processed in the absence (-) or presence of 5 or 10 µg of rmCD45. CD45 labeling on isolated neutrophils was analyzed by flow cytometry. (**A**) Representative flow cytometry plots of Ly6G^+^ neutrophils isolated from lungs of control PBS (top panel) or NTHi-infected mice (bottom panel). Unlabeled control mice were used to define positive populations (left). (**B**) Gating strategy for airway, blood or tissue neutrophils. (**C**) Flow cytometry plots of Siglec-F^+^CD11c^+^ resident alveolar macrophages following *in vivo* labeling with i.t. anti-CD45 alone (left) or i.v. and i.t. antibodies. Data are representative of two experiments with n = 3 – 5 mice per group. Abbreviations: NTHi, non-typeable *Haemophilus influenzae*; rmCD45, recombinant murine CD45; i.v., intravenous; i.t., intratracheal; FITC, fluorescein isothiocyanate; APC, allophycocyanin.

**Supplementary Figure 4: Neutrophils exhibit location-specific gene expression signatures after NTHi infection.** Mice were intranasally infected with NTHi (5 x 10^7^ CFU/mouse) and administered with i.v. or i.t. anti-CD45 antibodies at 48 h post infection followed by scRNA-seq analysis. (**A**) UMAP of Ly6G^+^ lung neutrophils subclustered based on gene expression data. (**B**) Analysis of CD45 labeling in transcriptional subclusters with anti-CD45.2-labeled airway neutrophils depicted in blue, anti-CD45-labeled blood neutrophils in green and unlabeled parenchymal neutrophils in orange. Data are representative of one sequencing experiment with n=3 mice per group.

**Supplementary Figure 5: Intravascular and airway immune cells can be distinguished by compartmental CD45 labeling in an LPS-induced acute lung inflammation model.** Mice were intranasally challenged with LPS (0.3 mg/kg) or PBS as a control. 24 h after challenge mice were administered with 2 µg anti-CD45-FITC (clone 30-F11) and anti-CD45.2-APC (clone 104) intravenously or intratracheally, respectively. Unbound antibodies were neutralized with 10 µg of rmCD45 and BAL and lungs were isolated. CD45 labeling and PD-L1 surface expression on Ly6G^+^CD11b^+^ neutrophils were analyzed by flow cytometry. Cells were categorized as airway, blood or tissue cells based on their respective CD45 label. (**A**) Representative flow cytometry plots of Ly6G^+^ lung neutrophils from lungs of control PBS (top panel) or LPS-treated mice (bottom panel). Unlabeled control mice were used to define positive populations (left). (**B**) Representative flow cytometry histogram plot of PD-L1 cell surface expression on airway (blue), parenchymal (green), and blood neutrophils (red). Flow cytometry data are representative of one experiment with n = 3 mice per group. Abbreviations: LPS, lipopolysaccharide; i.v., intravenous; i.t., intratracheal; FITC, fluorescein isothiocyanate; APC, allophycocyanin.

**Supplementary Table 1: TotalSeq-B and fluorescent antibodies.**

| **Target** | **Clone** | **Barcode** | **Sequence** | **Supplier** | **Cat#** |
| --- | --- | --- | --- | --- | --- |
| CCR2 | SA203G11 | B0426 | AGTGCGATCTGCAAC | BioLegend | 150633 |
| CD3 | 17A2 | B0182 | GTATGTCCGCTCGAT | BioLegend | 100257 |
| CD4 | RM4-5 | B0001 | AACAAGACCCTTGAG | BioLegend | 100573 |
| CD8 | 53-6.7 | B0002 | TACCCGTAATAGCGT | BioLegend | 100783 |
| CD11b | M1/70 | B0014 | TGAAGGCTCATTTGT | BioLegend | 101273 |
| CD11c | N418 | B0106 | GTTATGGACGCTTGC | BioLegend | 117359 |
| CD19 | 6D5 | B0093 | ATCAGCCATGTCAGT | BioLegend | 115563 |
| CD24 | M1/69 | B0212 | TATATCTTTGCCGCA | BioLegend | 101847 |
| CD25 | PC61 | B0097 | ACCATGAGACACAGT | BioLegend | 102067 |
| CD45 | 30-F11 | B0096 | TGGCTATGGAGCAGA | BioLegend | 103161 |
| CD45.2 | 104 | B0157 | CACCGTCATTCAACC | BioLegend | 109859 |
| CD64 | X54-5/7.1 | B0202 | AGCAATTAACGGGAG | BioLegend | 139329 |
| CD88 | 20/70 | B1042 | GCAGTCCTACATTGA | BioLegend | 135821 |
| CD103 | 2E7 | B0201 | TTCATTAGCCCGCTG | BioLegend | 121445 |
| CD172a | P84 | B0422 | GATTCCCTTGTAGCA | BioLegend | 144043 |
| CX3CR1 | SA011F11 | B0563 | CACTCTCAGTCCTAT | BioLegend | 149045 |
| F4/80 | BM8 | B0114 | TTAACTTCAGCCCGT | BioLegend | 123155 |
| Ly-6C | HK1.4 | B0013 | AAGTCGTGAGGCATG | BioLegend | 128053 |
| Ly-6G | 1A8 | B0015 | ACATTGACGCAACTA | BioLegend | 127659 |
| MERTK | 2B10C42 | B0565 | AGTAGAGCAACTCGT | BioLegend | 151525 |
| MHC II | M5/114.15.2 | B0117 | GGTCACCAGTATGAT | BioLegend | 107657 |
| PD-1 | RMP1-30 | B0004 | GAAAGTCAAAGCACT | BioLegend | 109125 |
| Siglec-F | S17007L | B0431 | TCAATCTCCGTCGCT | BioLegend | 155517 |
| TCRβ | H57-597 | B0120 | TCCTATGGGACTCAG | BioLegend | 109261 |
| XCR1 | ZET | B0568 | TCCATTACCCACGTT | BioLegend | 148231 |
| Rat IgG1, κ | RTK2071 | B0236 | ATCAGATGCCCTCAT | BioLegend | 400465 |
| Rat IgG2a, κ | RTK2758 | B0238 | AAGTCAGGTTCGTTT | BioLegend | 400581 |
| Rat IgG2b, κ | RTK4530 | B0095 | GATTCTTGACGACCT | BioLegend | 400689 |
| Hamster IgG | HTK888 | B0241 | CCTGTCATTAAGACT | BioLegend | 400979 |
| Mouse IgG1, κ | MOPC-21 | B0090 | GCCGGACGACATTAA | BioLegend | 400185 |
| Mouse IgG2a, κ | MOPC-173 | B0091 | CTCCTACCTAAACTG | BioLegend | 400291 |
| Mouse IgG2b, κ | MPC-11 | B0092 | ATATGTATCACGCGA | BioLegend | 400379 |
| **Target** | **Clone** | **Conjugate** | **Dilution** | **Supplier** | **Cat#** |
| CD11b | M1/70 | BV650 | 1:2000 | BioLegend | 101259 |
| CD16/CD32 | 2.4G2 | - | 1:200 | Bio X Cell | BE0307 |
| CD45 | 30-F11 | FITC | i.v. or 1:800 | BioLegend | 103108 |
| CD45.2 | 104 | APC | i.t. or 1:800 | BioLegend | 109814 |
| Ly-6G | 1A8 | BUV661 | 1:2000 | BD | 741587 |

i.v., intravenous

i.t., intratracheal
