# Supplementary figures and images for: Differential in vivo labeling with barcoded antibodies allows for simultaneous transcriptomic profiling of airway, lung tissue and intravascular immune cells
Source: Front Immunol. 2023 Nov 29;14:1227175. doi: 10.3389/fimmu.2023.1227175 (PMC10716273; doi:10.3389/fimmu.2023.1227175)

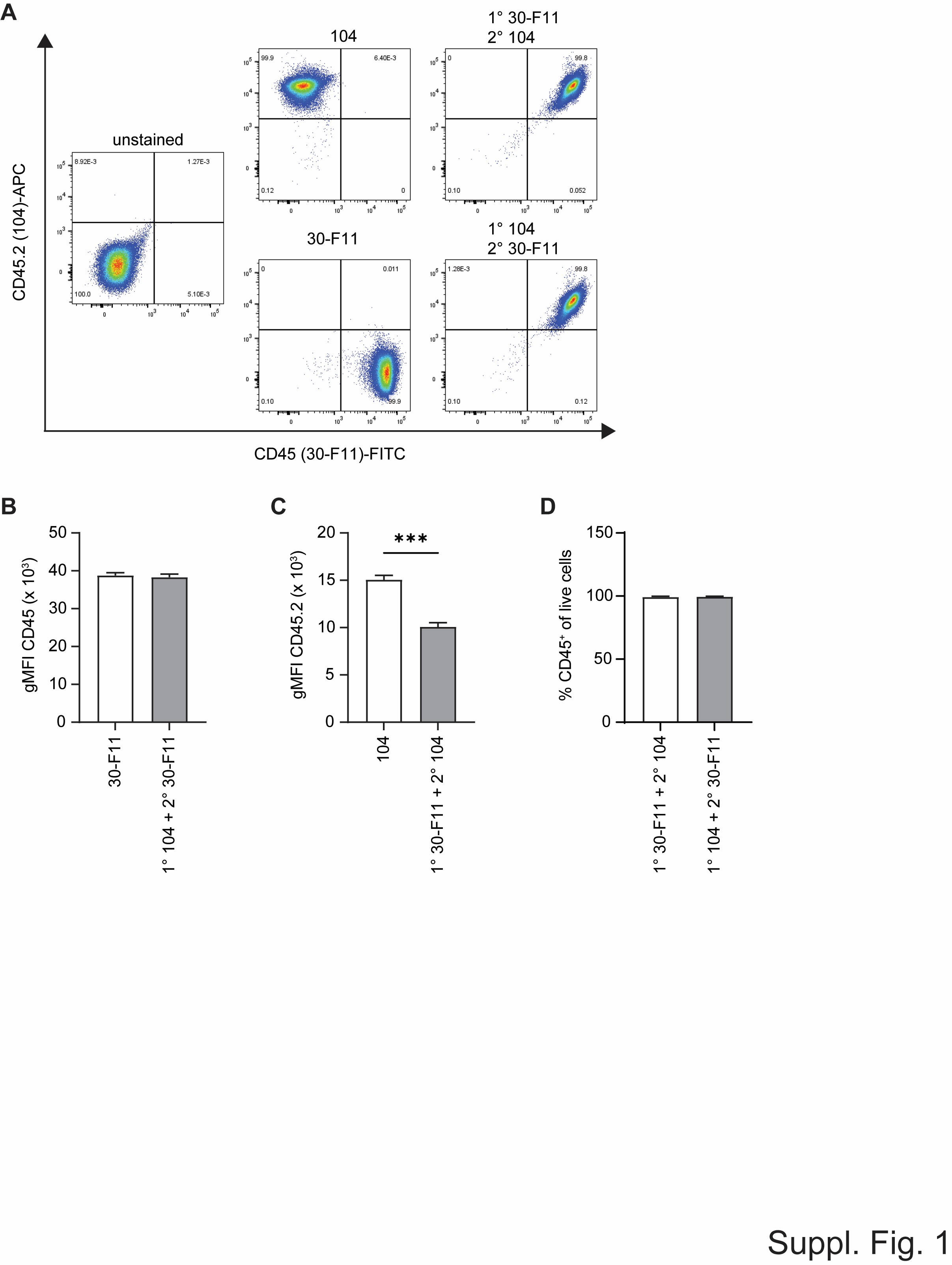

Supplement: Supplementary file 2 [file Image_1.jpeg]

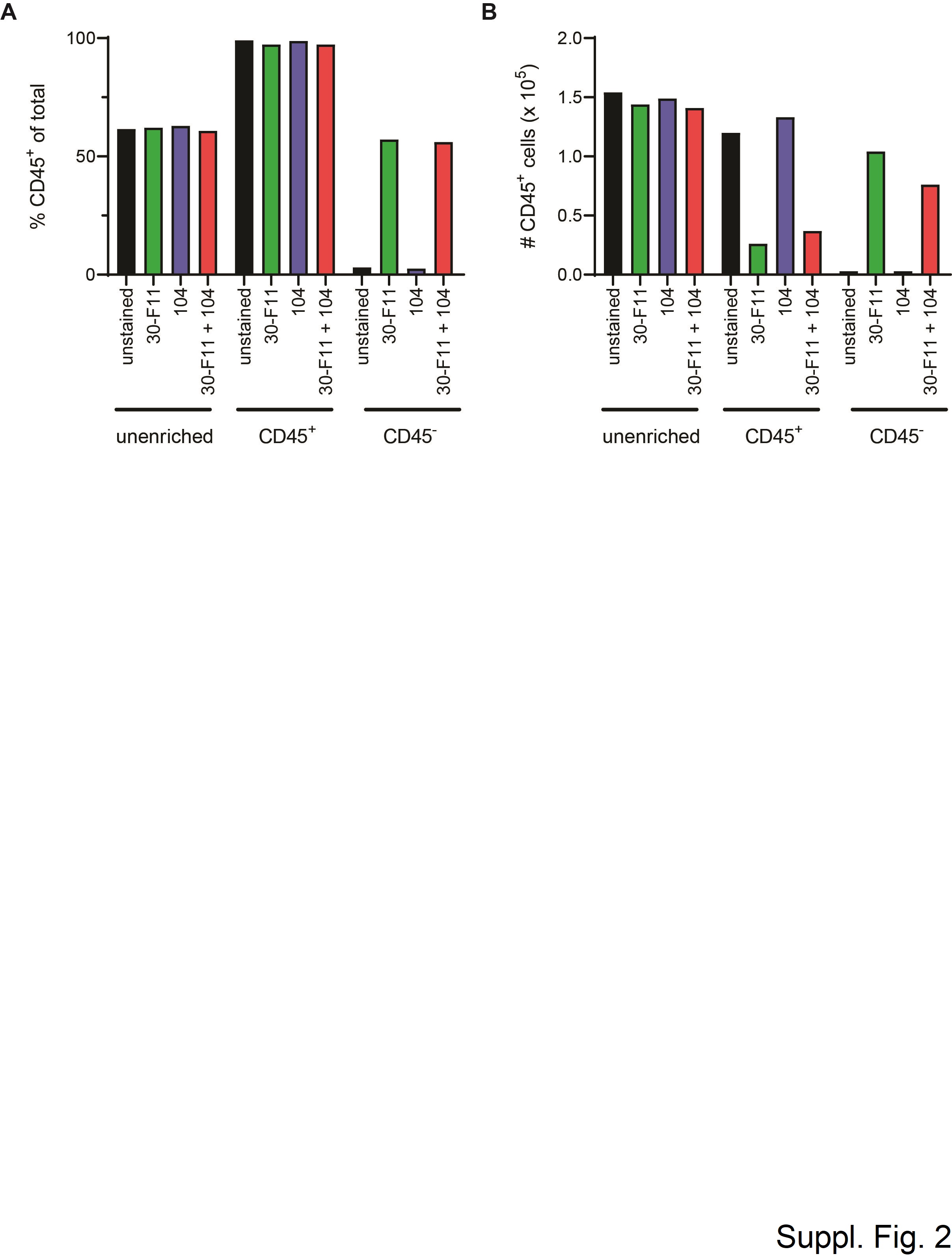

Supplement: Supplementary file 3 [file Image_2.jpeg]

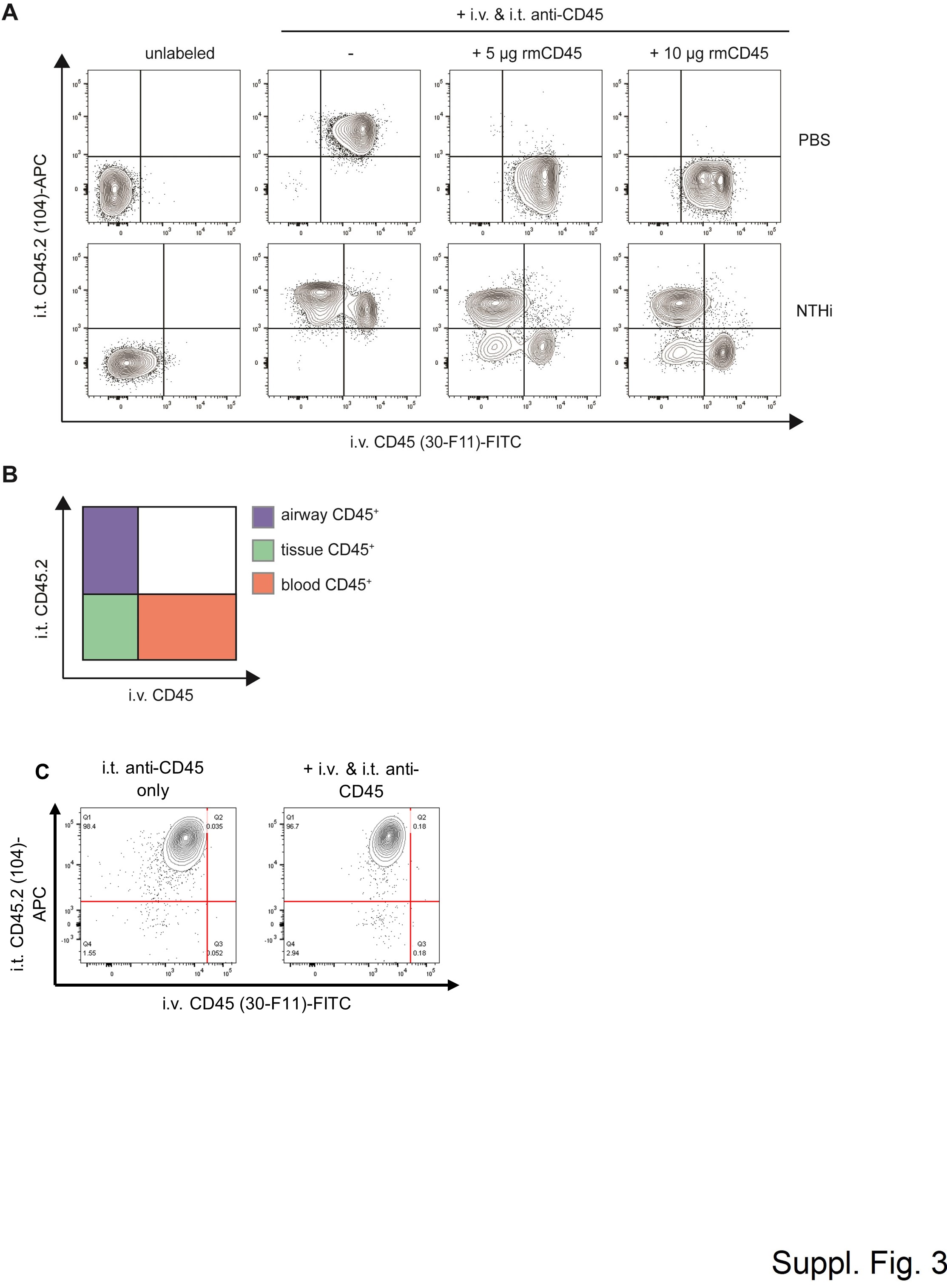

Supplement: Supplementary file 4 [file Image_3.jpeg]

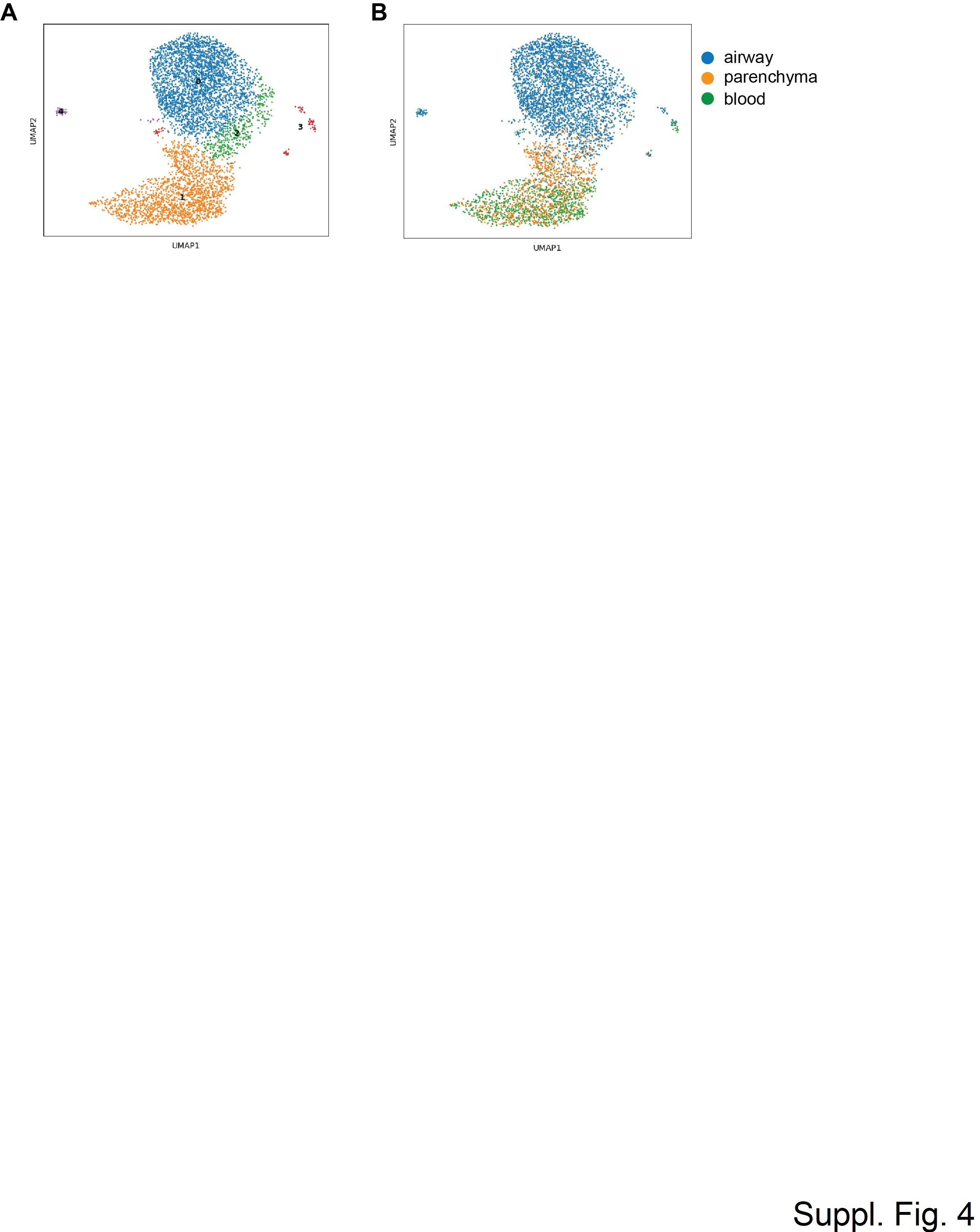

Supplement: Supplementary file 5 [file Image_4.jpeg]

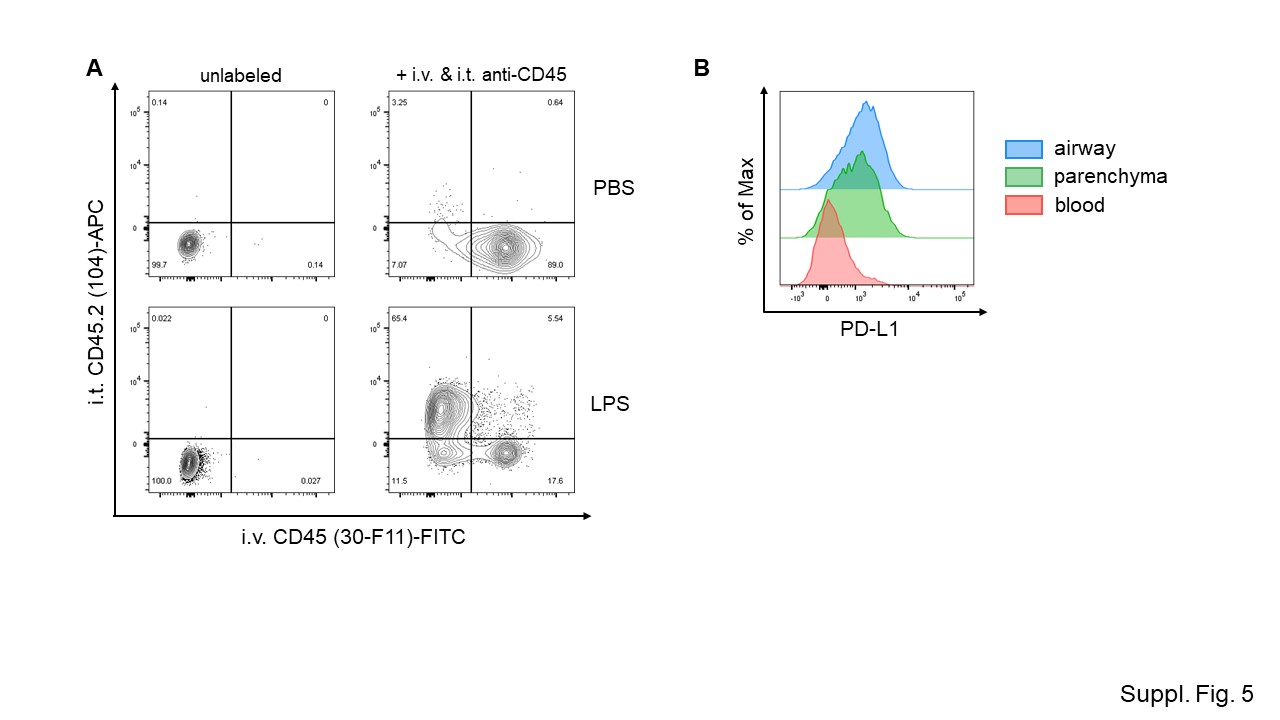

Supplement: Supplementary file 6 [file Image_5.jpeg]
